# Brief Notices of the Measles, Which Prevailed at Circleville, O. in the Spring of 1829

**Published:** 1829

**Authors:** John Cook Bennett


ART. VI.—*Brief notices of the Measles which prevailed at Circleville, O. in the spring of* 1829. BY DR. JOHN COOK BENNETT.

In the months of March, April, May and June, the measles prevailed in Circleville and its vicinity; chiefly, however, in the town. They affected every class of inhabitants—negroes not excepted. Not a few who had previously had the disease were reinfected and passed through all stages of the complaint with the usual symptoms. Even during the epidemic to which I refer, there were examples of its occurring twice in the same individuals, who, moreover, had experienced the disease several years before. I have not before known this malady so malignant.

*Symptoms.*—The disease commenced with a hoarseness, accompanied with a harsh dry cough, and uneasy or difficult respiration. Some cases began with a chill of short duration. The eyelids were tumefied, and the vessels of the conjunctiva became turgid and inflamed. The cheeks were frequently wet with a flow of acrid tears, and the nostrils were surcharged with acrid serum, which irritated their lining membrane to such a degree as to occasion an almost incessant sneezing. A violent cephalalgia and excessive headach were, generally, amongst the symptoms. The rash, which was "in crimson, stigmatised dots, grouped in irregular circles or crescents," generally made its appearance upon the fourth day, but it would frequently appear on the second day, and in two cases, which came under my notice, it did not show itself till the sixth. One of these cases terminated favourably, but the other person died of congestion of the lungs. A general scarlet flush often accompanied the pathognomic dots. The sooner the efflorescence made its appearance, the slighter and more favourable in most cases was the attack; but in some few instances where the rash made its appearance even upon the second day, the patient died. Many cases terminated on the fourth day, but if the disease passed the fourth it invariably continued until the ninth or afterwards. About a day prior to the termination of the disease, whether upon the fourth or ninth day, the redness diminished, and there was a branny desquamation of the cuticle, over the affected surface, leaving more or less discoloration of the skin. When the disease terminated favourably on either the fourth or ninth days, as these were the critical days, no trace of it remained: but when it terminated unfavourably, and not in death, in some cases sore eyes continued for some weeks, and in others where there was a predisposition to phthisis, that malady or some other pneumonic affection succeeded.

All the cases of death were from hydrocephalus or congestion of the lungs, consequent upon an aggravated state of the symptoms. In children it was hydrocephalus, in adults it was pulmonary congestion. In most cases the catarrhal fever was very high. There was no intermission of fever from the commencement to the termination of the disease, but a very slight remission each day.

*Treatment.*—We hold that fever is necessary to such an extent as to throw the virus upon the surface; but when it passes that point it endangers life. As the pyrectic action was generally high toned, our remedies were antiphlogistic. As it regards blood-letting in measles we cannot agree with Dr. Good, toto cælo, owing, perhaps, to the circumstance of our western diseases requiring much more active depletion than European diseases. When called to treat a patient labouring under rubeola, I commenced with copious blood-letting, with the design of reducing the pyretic action so as to excite a healthy action on the skin. This plan was attended with signal success, as only one patient died who was bled. It was observed that those who submitted to blood-letting had the disease much lighter than those who did not. Bleeding was resorted to in any stage of the disease when the symptoms seemed to demand it. The high toned pyrectic action would frequently demand it in the first stages, and the pneumonic inflammation in the last. There did not appear to be any danger of typhous symptoms supervening upon the abstraction of blood, as the symptoms participated more of synocha than of synochus. If the fever had proved a synochus there would have been danger of exhausting the patient by blood-letting, and so producing a fatal termination in typhus; but the fever, was catarrhal, and participated much of synocha, and required active depletion to secure a safe or favourable issue. The abstraction of blood was in proportion to the urgency of the symptoms, from twelve to thirty-six ounces. Phlebotomy was repeated pro re nata. Where the bowels were greatly disordered, and the primæ viæ required active evacuation, I used submurias hydrargyri from forty-two to eighty-four grains at a dosé. The 84 grain dose would produce no greater number of evacuations than the 42 grain doses, but each evacuation would be more copious. Calomel is surely a safe remedy, but it must be given in doses sufficiently large to work itself off, to avoid danger. It is the small dose, and not the large, that is attended with dangerous consequences, by remaining in the system so long as to produce a troublesome salivation, and it is these small doses that generally produce mercurial diseases. In ordinary cases, however, I used supertartrate of potash and flowers of sulphur, a.a. ʒi. mixed every three hours, until the *primæ viæ* were sufficiently evacuated. This was likewise used in some cases to promote the eruption. Emetics, of four grains of tart. antimo. mixed with ipecacuanha, sixteen grains, proved useful on the incursion of the disease, but subsequent to venesection.— They were of use in almost every stage of the disease. Where the cough or dyspnæa were troublesome, and where diaphoretics were necessary to produce a moisture on the skin, Dower's powder, oxymel of squills, small doses of pulvis antimonialis, a warm decoction of serpentaria virginiana and valerian combined, saffron tea, &c. were given as the symptoms seemed to require the one or the other. They were all used in turn with the happiest results. The doses were generally large. When the febrile symptoms were high and the thirst insupportable, cold water was allowed the patient as often as he desired it. It was used as a refrigerant beverage with good effect. The drinks were generally given hot, antecedent to the eruption, but after the efflorescence was fully established, cold water and other cold drinks were allowed. And in some cases cold refrigerent drinks were given anterior to the eruptions having appeared. The sick were generally kept cool in well ventilated rooms, but the currents of cold air were not suffered to pass immediately over the body of the patient. It was frequently necessary to wash the eyes with warm milk and water, in the morning before they could be opened, and to continue the bathing several times a day in order to remove the puriform discharge. It was necessary to wash the mouth likewise, so as to remove the sordes.— In some cases the whole body was bathed in warm milk and water. It will be unnecessary to enumerate other remedies, either of a general, specific, or topical nature, as the common remedies adapted to the symptoms were given as they were demanded or required. The food was light and vegetable, and usually in a liquid form. A diarrhœa would often supervene upon the subsidence of the eruption which always produced a favourable crisis. The diarrhœa was seldom corrected, and when corrected it was very cautiously done.—Pediluvium was often employed to produce perspiration. Injections were used very freely in all stages of the disease, and the happiest results were attendant upon their administration.

*October*, 1829.

